# Effects of a Home Literacy Environment Program on Psychlinguistic Variables in Children from 6 to 8 Years of Age

**DOI:** 10.3390/ijerph18063085

**Published:** 2021-03-17

**Authors:** Marta Romero-González, Rocío Lavigne-Cerván, Marta Sánchez-Muñoz de León, Sara Gamboa-Ternero, Rocío Juárez-Ruiz de Mier, Juan Francisco Romero-Pérez

**Affiliations:** Department of Developmental and Educational Psychology, University of Malaga, 29071 Málaga, Spain; marta_rg@uma.es (M.R.-G.); msanchez@neuropsipe.com (M.S.-M.d.L.); sgamboa@neuropsipe.com (S.G.-T.); rjrm@uma.es (R.J.-R.d.M.); romepere@uma.es (J.F.R.-P.)

**Keywords:** HLE, phonological awareness, reading decoding, vocabulary, oral narrative comprehension, elementary education

## Abstract

(1) Background: the objective of this study was to improve certain psycholinguistic and cognitive skills that are fundamental to the development of the reading process, such as phonological awareness, reading decoding, vocabulary and oral narrative comprehension, through the introduction of an Home Literacy Environment Active (HLE(A)) program that involved 54 participants enrolled in the first and second grades of elementary school (from 6 to 8 years old) in the city of Malaga area. (2) Methods: The central task of the intervention program was for the child to read aloud to an adult in the family for between 10 and 15 min, four days per week. In addition, the school students were evaluated on four occasions, at the beginning and end of each academic year, using the Batería de Evaluación de los Procesos Lectores Revisada, Test para la Detección de la Dislexia en niños and Escala Weschsler de Inteligencia instruments. (3) Results: the results demonstrated the efficacy of the HLE(A) program in the improvement of psycholinguistic and cognitive variables measured and, consequently, to an improvement in reading learning and cognitive development. Ultimately, the scientific literature on the subject and the data from the study led us to suggest that it would not only be beneficial for HLE(A) programs to be instituted in early childhood education stage (up to 6 years of age), but that they should be continued after age 6, in elementary education.

## 1. Introduction

Reading is an extremely complex, constructive and inferential process, in which the experience of the individual constitutes a determining factor in the reconstruction of meanings and the understanding of language, as they continuously create and check hypotheses throughout the reading act [1,2]. The importance of reading does not reside in a single aspect: because of it, human beings are able to acquire and modify schemes of knowledge, to train and enhance basic cognitive processes, to activate emotions and feelings, etc. That is, through the act of reading, the individual improves their cognitive and affective development, and, insofar as this supports learning, this higher psychological process and the mastery thereof have, over the years, aroused great interest in the educational field.

Owing to advances in cognitive psychology, research into reading and learning has been greatly expanded, allowing the determination of some of the early skills in reading development, also called literacy skills. These comprise a series of psycholinguistic processes that enable and enhance the learning of the reading act [3], such as phonological awareness, reading decoding, vocabulary, and oral narrative comprehension, all of them acquired at an early age.

It is worth highlighting the role played by these skills in the performance of reading tasks. Kim et al. (2015) [4] state that enhancing these competences benefits the subsequent evolution of the reading system, which is why acquiring them is fundamental [5] and why we may understand them as emergent literacy skills. In the scientific community, there is clear agreement that the above skills are among the most fundamental pillars supporting access to reading [6].

More specifically, among the psycholinguistic skills that most influence reading performance is phonological awareness, being the knowledge of the phonology of language that allows the identification, segmentation, and manipulation of units of speech as sublexical elements, that is, phonemes and syllables [7]. Phonological awareness influences and enhances learning of the rules of grapheme-phoneme conversion and vice versa. Thus, the greater the individual’s phonological knowledge, the easier it will be for them to associate speech sounds with corresponding graphemes [7,8,9,10]. As a consequence, this skill is regarded as an element that discriminates between good and bad readers or writers, as well as a predictor of future reading performance [11,12].

Phonological awareness is highly related to the process of decoding and identifying written language, a competency fundamental to reading, which requires the association of graphic representations with their respective sounds, on each occasion more rapidly and efficiently, until the process is automated. In this regard, a greater command of phonological awareness facilitates the establishment of such relationships and vice versa.

The decoding and identification of written language is understood as a specific process within the reading system that consists of recognizing isolated and/or combined graphemes in more complex units, syllables and words. This is done by identifying and assembling the sounds or phonemes of the different elements and/or performing a visual analysis of the word or part of it to access an orthographic form that is associated with a sound and, generally, to access its meaning. This skill is essential to the ability to read and, even more importantly, its automation strengthens its relationship with reading comprehension and vice versa. For example, reducing time and cognitive resources during identification allows a greater exploitation of the above competences during the reading comprehension process, such that one goes from thinking in order to decode to thinking about what is being decoded.

However, in order to establish the aforementioned relationship and to automate the identification of written language, it is essential that the reader possesses the necessary and appropriate lexicon for the task. Herein, one of the reasons why vocabulary constitutes another psycholinguistic skill of great importance to the development of the reading system.

A high level of vocabulary results in superior performance in the set of competences that comprise oral language [13]. In this respect, mastery of the lexicon not only facilitates speech comprehension, but also contributes to greater fluency in speech, enriching one’s expressive language [14]. This explains, more specifically, how the individual acquires a better oral and written understanding of topics that they know in depth: they possess a broader vocabulary in relation to the subject in question, which has an effect on their ability to structure and express ideas and knowledge concerning the topic. The same is not true in the case of subjects not mastered by the individual or in which the individual has less experience. Consequently, it may be assumed that a high level of vocabulary will be associated, in older age groups, with better access to the lexicon during the decoding of written language and with a rapid and efficient retrieval of the form and meaning of the word that does not require high expenditure of cognitive resources and that, therefore, favors automation of the identification process. In this regard, it has been shown that, over time, a greater breadth and depth of lexical knowledge leads not only to increased naming speed, but also to improved reading comprehension [3,15,16,17]. In another regard, on many occasions, oral narrative comprehension entails the identification of the meaning of the word on the basis of the narrative context in which it is framed, facilitating the use and adaptation of the optimal lexicon. Above all, it is important to highlight the role of oral language as the individual begins to learn to read, when they do not yet have a large vocabulary: it increases knowledge of words and their meanings, thereby benefiting reading comprehension. As noted in the work of Cerasari et al. (2015) [18], greater exposure to storytelling during childhood is generally associated with better oral language in adulthood and, more specifically, with the comprehension and production of oral narratives. Therefore, it is expected that the listening to and oral comprehension of stories during early childhood will enhance the individual’s development of knowledge relating to the structures of stories, syntactic processing, and identification of the most relevant aspects, etc., benefiting later reading comprehension and written expression.

The importance of all the aforementioned skills has led researchers to the study of literacy programs and the development of activities that promote reading. Early literacy programs are emerging—applied in nursery schools—in which the focus is on the linguistic processes that comprise oral and written communication [3].

As stated by Díaz and Soto (2019) [19], the creation and development of a reading habit requires the intervention of numerous agents and contexts, with the family as one of the factors in promoting the appropriate introduction of a reading routine, in addition to encouraging the requisite appetite and motivation. In turn, there are numerous references to or research studies based upon the construct that the present authors call the home literacy environment (HLE). This expression refers to the quantity and quality of resources and skills that families possess and which permit them to offer their children new learning opportunities [20,21]. According to numerous authors, such as Inoue et al. (2018), Sénéchal and Young (2008), and Wiescholek et al. (2018) [22,23,24], among others, two dimensions of HLE can be distinguished: (i) passive HLE (hereafter HLE (P)), which relates to the parents’ attitude towards reading and how they function as reading role models, and; (ii) active HLE, which also encompasses the set of activities aimed at developing literacy skills that are carried out by families with their children. As a consequence, active HLE programs (hereafter HLE (A)) seek to make use of family relationships and involvement in the activities—focused on the promotion of language skills—that are carried out with children at an early age, with the objective of strengthening the reading habit, as well as the acquisition and development of oral language [14]. Therefore, taking into account the relevance of emergent literacy skills, the variables traditionally subject to intervention in HLE programs have been those that are directly related to performance and knowledge of oral language and, subsequently, to reading [4,25,26,27].

In this respect, various researchers have analyzed the positive effect of HLE (P) on language development in preschool children [28] and on their subsequent reading performance [25]. Specifically, Kim et al. (2015) [4] conducted a study in which the main objective was to measure the effect of general aspects of the HLE on phonological awareness, word decoding, and vocabulary in a broad sample of 6050 children, collecting data from the HLE when the children were 2 years of age and assessing performance in psycholinguistic skills at 4-and-a-half years of age. To do this, they used the modified Peabody Picture Vocabulary Test [29], the MacArthur Communicative Development Inventory [30], the Test of Phonological and Print Processing [31], and an ad hoc questionnaire for parents, designed to measure four HLE characteristics (number of books in the home, shared reading, storytelling by parents to children, and frequency with which families sing with their children). In their study, the authors found positive correlations, on the one hand, between HLE and vocabulary and, on the other, between vocabulary, phonological awareness, and word decoding. As might have been expected, the improvement of the four aforementioned HLE characteristics benefited the child’s vocabulary level, as well as their phonological awareness and word recognition skills.

After a review of the scientific literature on the subject, it has been found that HLE programs have different characteristics and focus on different types of activities [32]. It has been mentioned, the difference between active and passive HLE [26]; however, we have not delved into what activities are typically included in HLE (A) programs. One of the most frequent reading tasks at early ages is shared reading [24], specifically, dialogic reading [33]. However, Sénéchal and Young (2008) [24] in their meta-analysis analyze the effect of child-to-parent reading, tutoring and carrying out concrete activities to teach their children to read at home (parents working with their children on grapheme-phoneme association activities) and shared reading on literacy skills (not including emergent skills associated exclusively with oral language, focusing on decoding and reading fluency) in kindergarten children. The researchers observed that both child-to-adult reading and tutoring specific activities had positive effects on children’s literacy skills, unlike shared reading (note that oral language skills were not included). In addition, they found that parental involvement had a mean weighted effect size on their children’s literacy skills (Cohen’s *d* = 0.65). All this leads us to suppose that, at later ages (between 6 and 8 years old), when children have greater reading skills and fluency, the reading activity from the child to the adult becomes even more relevant. Especially if the adult guides, corrects errors and encourages reading comprehension through questions about the text read.

Meanwhile, Mora-Figueroa et al. (2016) [34] assessed performance in reading decoding and listening comprehension, among other variables, in a sample of 206 students (between 6 and 7 years old)—by means of tasks, drawn from the PROLEC-R test, comprising the reading of words and pseudowords and listening comprehension [35]—before and after the application of an HLE (A) program that consisted of shared reading tasks and the child reading aloud (three times per week in both cases, for 10 or 15 min), for 12 weeks. After analyzing the data, the researchers found a significant improvement in the reading and oral comprehension performance of the group of participants who undertook the training (a group of 156 children) in comparison to the group without any intervention (a control group of 50 students).

Niklas and Schneider (2014) [14] conducted a study with 125 children, aged 4:10 to 6:6 years, with the objective of measuring the effect of an active, non-intensive HLE program (applied over the course of a school year; 8 months) on children’s performance in letter identification, naming speed, and vocabulary tasks. In this program, two phases were defined: the first consisted of a set of three interviews and sessions with the families—over the course of one week, for 40 min in each instance—detailing the relevance of active HLE and providing suggestions on how to improve it; the second consisted of carrying out a shared reading activity for 20–30 min, in which the father or mother had to read a story to their child and encourage interaction in the process (for example, creating conversations about accompanying illustrations, asking questions about it, etc.), following which they received corrections and recommendations from an expert observer. A control group was established in addition to the three intervention groups (Group 1, which only undertook the first phase; Group 2, which carried out the second phase; and Group 3, which carried out the entire intervention program). Moreover, the collection of data during the evaluations, before and after the application of the program, was achieved using the Revised Vocabulary Test for 3- to 5-year-old children [36], the Columbia Mental Maturity Scale [37], and an ad hoc questionnaire for parents, designed to gather information relating to other HLE variables (number of books, number of literacy activities). It was found that 86% of the parents who participated in the second phase subsequently began to carry out the literacy activity at least once per week and, focusing exclusively on the variables that this study addresses, the researchers found that the intervention groups obtained better results in the identification of letters and vocabulary. In regard to the latter variable, a significant difference was observed in Group 3, which completed both phases of the intervention, which completed both phases of the intervention, obtaining a medium effect size (Cohen’s *d* = 0.59).

Based on the previous intervention program, Niklas and Schneider (2017) [38] conducted a follow-up study, evaluating the previous sample (of 125 children) when they were between 5 and 6 years old. Specifically, they measured the effect of the active HLE intervention program—6 months following its application—on vocabulary and phonological awareness, which were assessed using the aforementioned tests in addition to two more tasks for measuring phonological awareness: eliminating the initial phoneme from a set of stimuli, from the Revised Vocabulary Test [36], and identifying the first sound of the word. The authors observed that the non-intensive intervention was sufficient for some of the benefits of the HLE (A) to endure over time. Specifically, they showed that the improvement in phonological awareness was maintained 6 months following the implementation of the program; the scores of Groups 2 and 3 (who carried out the second phase and the complete intervention, respectively) were higher, with an effect size of 0.46, and the same results were not obtained for vocabulary.

As a consequence of previous research into HLE, we carried out an investigation that aimed to demonstrate the efficacy of an HLE (A) program for the improvement of, principally, psycholinguistic skills and cognitive competences related to reading, in addition to the specific psychological processes of the reading system, variables associated with motivation to read in the youngest children, and the quality of their family relationships. However, due to the large number of variables assessed in the course of the investigation, only some of them were selected for the present study.

With this, we intend to contribute new data to science on the effects of an HLE (A) program on a series of psycholinguistic and cognitive variables in a sample of children aged 6 to 8 years. As mentioned, there are many studies that have focused on the beneficial effects of HLE on these types of variables in samples of children under 6 years of age [12,36,37,38,39,40], among others; however, there are fewer studies that seek to analyze these types of variables at older ages. Considering that these are variables that evolve, are related to and are affected by specific reading processes—such as decoding, which benefits from adequate phonological awareness and vice versa—we believe that they should also be studied in the early years of primary education (between 6 and 8 years of age). Especially in view of the latest national and international reports [41,42,43,44,45,46] that point out the need to incorporate -from the first years of compulsory education in Spain, specifically in Andalusia—study plans that include reading programs that contribute to the development of specific reading processes and that, as a consequence, increase the reading habit, enhance learning and reduce the school dropout rate.

The general objective was to improve certain fundamental skills in the development of the reading process, such as phonological awareness, reading decoding, vocabulary, and oral narrative comprehension, through the introduction of an HLE (A) program that involved a group of 54 participants enrolled in the first and second grades of elementary education (from 6 to 8 years old), at a colegio concertado (charter school; a state-funded but privately managed school) in the city of Malaga area.

Specific objectives were set as follows: (1) improve phonological awareness (phonemic segmentation, rhymes and verbal fluency), reading decoding (reading and reading without sense), vocabulary (semantic fluency, vocabulary and verbal comprehension), and oral narrative comprehension in the sample—through the application of an HLE (A) program—over the course of 9 months, (2) maintain the improvements in phonological awareness (phonemic segmentation, rhymes and verbal fluency), reading decoding (reading and reading without sense), vocabulary (semantic fluency, vocabulary and verbal comprehension), and oral narrative comprehension in the sample—arrived at through the application of an HLE (A) program—following 3 months of summer vacation, without training and; (3) improve phonological awareness (phonemic segmentation, rhymes and verbal fluency), reading decoding (reading and reading without sense), vocabulary (semantic fluency, vocabulary and verbal comprehension), and oral narrative comprehension in the same sample—through the application of an HLE (A) program—following a further nine months of intervention; when the participants are one year older than at the beginning of the study and are, therefore, in the next grade at school.

Accordingly, as a general hypothesis of the present study, it was expected that, upon the application of an HLE (A) program, certain fundamental skills in the development of the reading process would improve, such as phonological awareness, reading decoding, vocabulary, and oral narrative comprehension, in a group of 54 participants enrolled in the first and second grades of elementary education (from 6 to 8 years old), at a colegio concertado (charter school; a state-funded but privately managed school) in the city of Malaga area.

The specific hypotheses of the study were as follows: (1) it was expected that phonological awareness (phonemic segmentation, rhymes and verbal fluency), reading decoding (reading and reading without sense), vocabulary (semantic fluency, vocabulary and verbal comprehension), and oral narrative comprehension would improve in the sample if an HLE (A) program was applied over nine months; (2) it was expected that the improvements in the sample with regard to phonological awareness (phonemic segmentation, rhymes and verbal fluency), reading decoding (reading and reading without sense), vocabulary (semantic fluency, vocabulary and verbal comprehension), and oral narrative comprehension—achieved through the application of an HLE (A) program—would be maintained following 3 months of summer vacation without training and; (3) it was expected that phonological awareness (phonemic segmentation, rhymes and verbal fluency), reading decoding (reading and reading without sense), vocabulary (semantic fluency, vocabulary and verbal comprehension), and oral narrative comprehension would further improve in the same sample if an HLE (A) program was applied for an additional nine months. That is, when the participants were one year older than at the beginning of the study and, therefore, were in the next grade at school.

## 2. Materials and Methods

### 2.1. Participants

In order to select participants, a cluster sampling of students was carried out. The group of students selected for the study included 54 children (24 girls and 30 boys), divided into 2 groups (1A and 1B) of 27 participants on the basis of which teacher served as the students’ tutor (i.e., in charge of teaching the majority of subjects). It is to be noted that it was not possible to include a control group from the same school year owing to the conditions imposed by the families of the participants and the school, who expressed their desire to implement the HLE (A) program as a school reading plan, such that no student was excluded from it.

In carrying out the study, we had the collaboration of a state-funded educational center in the city of Malaga, with a middle- and upper-middle-class demographic and with parents who had training—in many cases at the university level—that facilitated the application of the HLE (A) program and the evaluation of its effect on psycholinguistic and cognitive variables, by controlling the interference of other variables such as cultural differences, lack of availability of resources, low linguistic and/or reader level of families, etc. Importantly, this study was carried out in accordance with the recommendations of the Faculty of Educational Sciences and the Regulations of the Ethical Committee on Experimentation at the University of Malaga. Further to this, the study complies with the requirements of Spain’s Ley Orgánica de Protección de Datos (Constitutional Law on Data Protection) 2/2018. Ethics approval was not required, in accordance with the guidelines and regulations of the University of Malaga.

In the selection of the sample, the requirements were as follows:

- Participants attended the above-mentioned educational center.
- Participants were enrolled in the first year of elementary education (at the beginning of the program).
- Participants were neither receiving nor had previously received specific reading acceleration programs.

Once the numbers and the type of participant had been decided on, the school informed students’ families. Subsequently, those who were interested proceeded to sign an “Informed Consent” document, detailing the justification for the study, its objectives, and its design.

### 2.2. Instruments

The following instruments (with psychometric guarantees of reliability and validity) were used to analyze performance in phonological awareness, reading decoding, vocabulary, and oral comprehension of narrations (see Table 1): Revisada, Test para la Detección de la Dislexia en niños (DST-J) [47], Batería de Evaluación de los Procesos Lectores (PROLEC-R) [35] and Escala Weschsler de Inteligencia (WISC-V) [48].

#### 2.2.1. Phonological Awareness

To assess phonological awareness, three tasks were selected from the DST-J instrument [47], a test designed for detecting and screening difficulties in learning to read in children from 6:6 to 11:5 years old; it provides a percentile scale, which allows the scores obtained from the individual evaluated to be set in the context of those from members of the general population with a typical development for the corresponding age. Of a particular relevance, this test can be administered by professionals in the school environment, in addition to psychologists, yet this must always be done on an individual basis.

The selected tasks were:

- Phonemic segmentation. Assesses the ability to identify, segment, and manipulate phonemes, isolated or combined. The child must divide the word into parts, eliminating some of the consonants or a complete syllable, or replacing them with others. The test is ended if errors are made in the first four items or following three consecutive errors, with a count taken of the correct answers. The maximum direct score is 8. The validity and reliability of the measure has been demonstrated in the literature, specifically, the test presents a Cronbach’s reliability coefficient of 0.94 [47].
- Rhymes. Assesses the individual’s ability to segment and identify the final group of phonemes in the word. Pairs of words are presented orally and the child must indicate whether or not they rhyme. A count is taken of successful pairings. The maximum direct score is 8. The validity and reliability of the measure has been demonstrated in the literature, specifically, the test presents a reliability coefficient of 0.73 [47].
- Verbal fluency. Assesses the ability to identify the initial phoneme “p” in words from their lexical repertoire, within a given time. The child must name, within one minute, the maximum number of words beginning with “p”, with a count taken of the correct answers. The maximum direct score is 25. The validity and reliability of the measure has been demonstrated in the literature [47].

#### 2.2.2. Decoding and Identification in Reading

To assess decoding, two tasks were selected from the DST-J test [47]:

- Reading. Assesses the individual’s ability to recognize and identify words from a list. As these words are not inserted into a text, it was decided in our study to name the variable “word reading”. The student must read a set of words, in a given time, with one point counted for each word correctly decoded. The maximum direct score is 120. The validity and reliability of the measure has been demonstrated in the literature [47].
- Reading without meaning. Assesses the individual’s ability to recognize words and pseudowords (such as Norbi, rather than Norgin) inserted into a text. The maximum score is 58; one point is counted for each correct word and two for each pseudoword. Furthermore, if more or less than a minute is taken to complete the task, a half point is penalized or rewarded for each second (up to a maximum of 10 points). It should be noted that the text used in this task (an excerpt from Lewis Carroll’s Jabberwocky in Spanish), despite including elements without meaning, is not without sense. For this reason, in the study, it was decided to name the variable “reading without meaning” and not “without sense”. The validity and reliability of the measure has been demonstrated in the literature [47].

#### 2.2.3. Vocabulary

To assess vocabulary, three tasks from two different instruments were used. On the one hand, from the DST-J test [47], the following task was selected:

- Semantic fluency. This measures the breadth of the individual’s vocabulary. The child must name, within one minute, words belonging to the semantic field of animals, with a count taken of correct answers. The maximum direct score is 25. The validity and reliability of the measure has been demonstrated in the literature [47].

On the other hand, two tests were selected from the WISC-V instrument [48], a scale evaluating cognitive abilities in children and adolescents from 6 to 16:11 years old, always administered on an individual basis. The scores of both tests were combined to form the verbal comprehension index and thereby transform the results of the assessed students into percentile scores, with the scales provided by the instrument, as in the case of the DST-J [47].

The tests were:

- Similarities. Assesses the child’s ability to abstract similarities and make generalizations based on two given concepts. In this test, the student must indicate which characteristics share the meanings of two different words named by the examiner. In total, the test comprises 23 items, to be rated from 0 to 2 depending on the appropriateness of the response. The maximum direct score is 46. The validity and reliability of the measure has been demonstrated in the literature, specifically, the reliability coefficients of the individual subtests range from 0.80 to 0.94, demonstrating high levels of internal consistency [48,49].
- Vocabulary. Assesses the child’s lexical knowledge, conceptual accuracy, and expressive ability. In this test, the student must name a series of visual stimuli and define the words presented in oral form by the assessor. In total, the test comprises 29 items (4 that constitute visual stimuli and 25, verbal stimuli). The maximum direct score is 54. The validity and reliability of the measure has been demonstrated in the literature, specifically, the reliability coefficients of the individual subtests range from 0.80 to 0.94, demonstrating high levels of internal consistency [48,49].

#### 2.2.4. Oral Narrative Comprehension

For the assessment of this variable, a task from the PROLEC-R instrument [35] was used. This test is administered on an individual basis and is used to evaluate reading processes: specifically, to diagnose learning difficulties in reading among children aged from 6 to 12 years. It should be noted that because the instrument does not provide scales that allow the scores to be transformed into percentiles, it was necessary to compare the direct scores of participants with the mean averages obtained from the sample used to prepare the test.

The task selected from PROLEC-R as follows:

- Oral comprehension. Assesses the individual’s ability to understand narratives read by the assessor. Following the presentation of each story (two stories are told), four questions are asked, with a count made of the correct answers. The maximum possible number of points is 8. The validity and reliability of the measure has been demonstrated in the literature, specifically, the test presents a Cronbach’s reliability coefficient of 0.67 [35].

### 2.3. Procedure

In order to successfully implement the project, the objectives and each of the phases of the study were explained to the management team at the educational center and to the teachers involved. Subsequently, they were put in charge of communicating the relevant information to the families, who signed their informed consent after agreeing to the study and the selection of the sample, as mentioned above.

#### 2.3.1. Evaluation Phase

Measurement of the variables was carried out by collecting data from the whole sample on four different occasions, at the beginning and end of both school years—each of which lasted 9 months—or, put in precise terms: (i) before the introduction of the program, (ii) after 9 months of training, (iii) after 3 months of vacation period (without intervention) and, (iv) after 9 further months of applying the HLE (A) program.

The sessions for testing individual students lasted approximately one hour. Two weeks were required to assess all participants on each of the four occasions for evaluation.

#### 2.3.2. Intervention Phase

This phase began with researchers explaining to teachers and to participants’ families the benefits and the implications of HLE, in addition to the procedure to be followed during the school year, through training sessions (two for teachers and two for parents). Specifically, the task that was carried out in the family context consisted of the child reading to the adult—for 10–15 min, four times per week—and the latter correcting any errors in reading fluency, posing questions and making comments about the text, and encouraging topics of conversation in the family home relating to the books included in the project.

To facilitate the implementation of the program and to provide reading material appropriate to the students’ curricular level, 54 books were purchased, which were chosen by the teachers of the experimental groups and which were exchanged by children every Friday at school, prior to the end of the day, so that each child had a different book every week.

In turn, the parents had to fill in a booklet designed to provide a daily record of the students’ reading and to permit more exhaustive monitoring: (i) daily, by the teachers and; (ii) every two weeks, by the research team (psychologists and psycho-pedagogues), except if the teachers had any doubts, which were reviewed beforehand. Attached within the same notebook were the different sections that parents had to fill in (date, title of the book, number of pages read, a commentary on the difficulties detected, if any, doubts, progress and signature) and, as a reminder, was a brief summary of the guidelines that should be followed by the adult during and after the child’s reading:

- Daily reading time: minimum 10 min; maximum 15.
- Read in a quiet place, free from distractions.
- The child must read aloud.
- The adult must be attentive to the reading and make corrections in a positive way when necessary.
- At the end, the adult should ask the child two or three questions about what has been read (for example: Who is the protagonist of the story? Why do they act like this? etc.).
- Fill in the sheet in the booklet each day and enjoy this moment of reading with your child.

### 2.4. Design and Data Analysis

The study design was quasi-experimental, seeking to find a relationship between the independent variable (HLE (A) program) and the dependent variables (phonological awareness, reading decoding, vocabulary, and oral narrative comprehension).

Statistical analyses were performed using the Statgraphics 18 and SPSS 24 programs. In order to ensure that the results were not conditioned by age, we worked with percentile scores—based on the population reference patterns provided by the test [47,48]—for three of the variables: phonological awareness, reading decoding, and vocabulary. However, in the case of the oral narrative comprehension variable, because it was not possible to obtain percentile scores using the PROLEC-R test, the direct scores were compared with the population means of the sample used to prepare the instrument [35]. Thus, all variables analyzed fulfilled the assumptions of normality, homogeneity, and homoscedasticity, for which parametric analyzes were performed.

In comparing the scores for each variable, obtained on four different occasions, either an analysis of variance (ANOVA) for repeated measures or a mixed-factor analysis was carried out, in which both intra-subject (experimentation factor) variables and inter-subject (group) variables were considered [50,51].

To detect improvements in performance on each variable, significant differences in the experimentation factor were examined. Then, in order to study whether the behavior of the variable was similar in both groups of participants and to rule out the influence of other variables, we verified whether there were significant differences in the group factor. Finally, in order to verify whether improvements were maintained or increased over the four evaluations, among all the variables, we examined the trend of the scores for the experimentation factor.

## 3. Results

All the variables measured in the study were conditioned by two factors (group and experimental). The group factor (inter-subject variables) was divided into two levels—1A and 1B—at which the HLE (A) program was applied. In turn, the experimental factor (intra-subject variables) comprised four levels, or occasions, of evaluation, over two school years: (i) Evaluation 1, before starting the intervention; (ii) Evaluation 2, after 9 months of training; (iii) Evaluation 3, after 3 months of vacation period (without intervention) and; (iv) Evaluation 4, after 9 further months of applying the HLE (A) program.

First, in order to verify the efficacy of the HLE (A) program in relation to the improvement of the variables, a repeated measures ANOVA analysis was performed and changes in the experimental factor were examined. Additionally, sphericity was verified using the Mauchly sphericity test and, in the event of rejection, the Huynh-Feldt correction was used. In this way, significant differences (*p*-value < 0.05) were detected in the mean responses for all the variables measured. Second, in order to check whether the behavior of the variables was similar in both groups, the differences between the groups and their interaction with the intervention were given study. Following the analysis, it can be stated that there were no significant differences (*p*-value > 0.05) between the two groups of students, nor was there any significant interaction between the groups and the intervention in any of the variables. In brief, the effect of the HLE (A) program—similar in both intervention groups—was confirmed, in: phonological awareness (rhymes, phonemic segmentation, and verbal fluency), reading decoding (word reading and reading without meaning), vocabulary (semantic fluency and verbal comprehension), and oral narrative comprehension (see Table 2).

Third, in order to verify whether improvements were maintained or increased over the four evaluations, we carried out a trend analysis of the experimental factor and checked whether the evaluation could be assumed to be different from the mean given the confidence intervals calculated (see Table 3). As expected, this analysis showed that all variables improved following 9 months of applying the HLE (A) program, that is, between the first and second evaluations. It was also confirmed that following 3 months of summer vacation (without intervention, between the second and third evaluations), despite the scores of all the variables being reduced—except for reading, including reading decoding, which increased—the improvements were maintained. Further to this, it was shown that following the application of the HLE (A) program for a further 9 months (between the third and fourth evaluations), the improvements persisted in all of the variables; however, the existence of two clearly differentiated patterns was observed.

**Figure 1:**
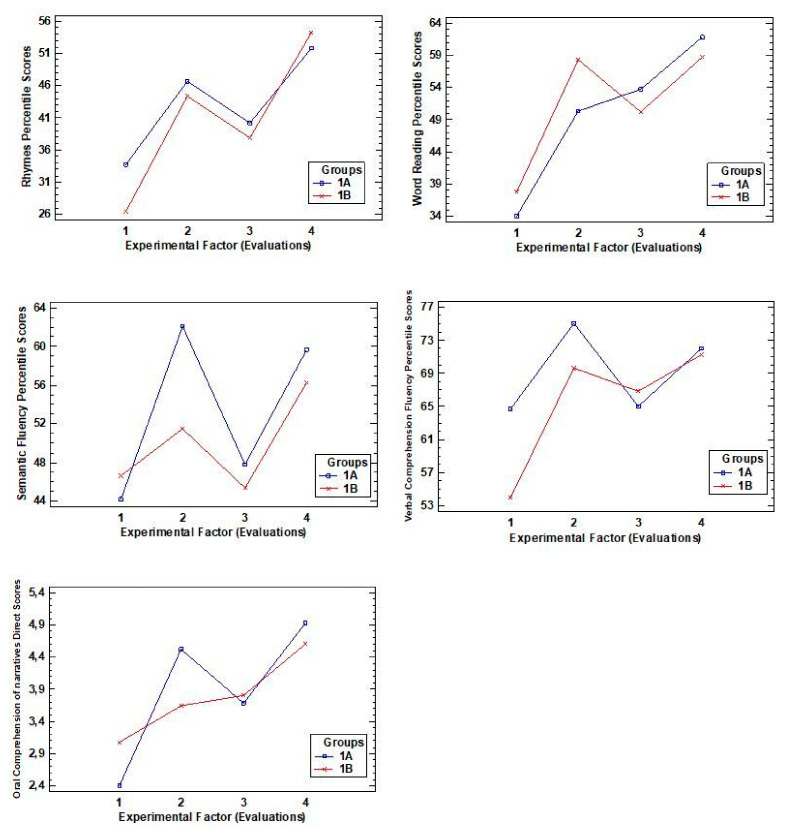
Graphs showing the interaction of the variables: rhymes (phonological awareness), word reading (decoding), semantic fluency and verbal comprehension (vocabulary), oral narrative comprehension.

The first pattern includes one variable associated with phonological awareness (rhymes), one for decoding (word reading), two for vocabulary (semantic fluency and verbal comprehension), and one for oral narrative comprehension (see Figure 1). Among these variables, it was found that: (i) all scores increased compared to the previous evaluation; (ii) improvements in rhymes (M = 53), reading (M = 60.28), and oral narrative comprehension (M = 4.76) increased, and; (iii) improvements in semantic fluency (M = 50.71) and verbal comprehension (M = 71.65) were maintained.

**Figure 2:**
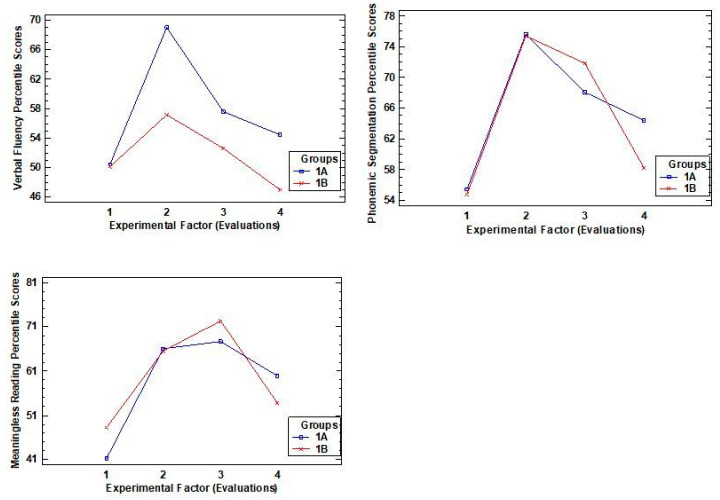
Graphs showing the interaction of the variables: verbal fluency and phonemic segmentation (phonological awareness), reading without meaning (decoding).

The second pattern, meanwhile, includes two variables associated with phonological awareness (verbal fluency and phonemic segmentation) and one with decoding (reading without meaning). Among these variables, it was observed that (see Figure 2): (i) all scores decreased compared to the previous evaluation; (ii) improvements were maintained in phonemic segmentation (M = 61.35) and reading without meaning (M = 56.94), while this was not the case for verbal fluency (M = 50.71), in which the mean scores obtained were similar to those at the beginning of the study. Thus, in the graphs representing the trends for these scores, a curve in the shape of a concave parabola was observed in the former two variables.

In summary, it can be stated that the HLE (A) program had a positive effect on all variables, with the exception of verbal fluency, which was included within phonological awareness. Although the results show different behaviors among some of the variables, evidenced in the pattern observed in the variables phonemic segmentation (phonological awareness) and reading without meaning (reading decoding), it was found that the effect of the HLE (A) program was maintained as a trend—over time—in most of the variables measured, these being: rhymes (phonological awareness), word reading (reading decoding), semantic fluency (vocabulary), verbal comprehension (vocabulary), and oral narrative comprehension.

## 4. Discussion

The general aim of this study was to improve the reading performance of the sample by applying an HLE (A) program; we were able to observe this intervention through the variables phonological awareness, reading decoding, vocabulary, and oral narrative comprehension. In accordance with our expectations, it can be stated, following analysis of the data, that between the first and last evaluation—that is, after two academic years—improvements were found in all variables, except in verbal fluency, for which scores did not increase, but were positioned around the population mean. Thus, in line with the results obtained by other authors, we were able to demonstrate the efficacy of the HLE (A) program for the improvement of psycholinguistic variables associated with reading [4,14,34,38].

Furthermore, it should be noted that similar behavior was detected in the results of both groups (1A and 1B) over the four instances of evaluation, in light of which we judge that the HLE (A) program had a positive effect on all participants, independent of the specific teachers in charge of teaching the different subjects. That is to say, the instruction that was given affected both groups in the same way, without interfering in the effectiveness of the program. Nevertheless, a decrease in the scores was observed following the vacation period because during this time no type of intervention was carried out, confirming the necessity of systematic training to the process of learning to read, and the importance and the influence of the family within this, as previously indicated by authors such as Díaz and Soto (2019) and Jiménez (2012) [19,20].

In respect of the variables included in phonological awareness (rhymes, verbal fluency, and phonemic segmentation), improvements were detected in all of these, between the first and last evaluations, with the exception of verbal fluency. These data bear a relationship to those found by Kim et al. (2015) and Niklas and Schneider (2017) [4,38]. The former referred, in their 2014 study, to a better performance in phonological awareness in those children who inhabited a more enriching HLE. Meanwhile, Niklas and Schneider (2017) [38] observed an improvement in phonological awareness, resulting from the application of an HLE (A) program, that was maintained over time until six months following the end of the intervention. However, although this study demonstrated that an HLE (A) program with a focus on reading tasks had a positive effect on phonological awareness—specifically, on rhymes and phonemic segmentation—it is appropriate to bear in mind that, as is commonly found in the learning and development of reading, the evolution of the phonological skills measured is not homogeneous in all cases, nor at all ages. This is at least in part because the management of resources and the use of different skills vary during the learning of the reading act, owing to the complexity of the process.

Specifically, as indicated by De la Calle et al. (2018) [52], syllabic awareness appears before phonemic awareness in transparent languages such as Spanish, such that children learn to perform rhyme—through analogies—from an early age. In this way, the mastery of rhyme is rendered a simpler and a quicker task, particularly given that children are exposed to rhymes on a continuous basis (adults play word games to form rhymes, and some stories and songs include them, etc.). Yet, this does not happen in the same way with phonemic segmentation. In this study, a strong improvement in scores could be observed during the first academic year, when children work more specifically on phonological awareness and reading; however, following the summer months there was a decrease in the improvements obtained after the first 9 months of intervention. This is due to the fact that, from the age of 7, children are in the process of automating reading—specifically, decoding—making greater use of the lexical path and focusing less attention on the development of phonological skills, committing a greater number of errors in the segmentation and manipulation of sublexical elements.

The same evolution was displayed by the sample as regards verbal fluency, although differing to the degree that similar results were obtained in the first (M = 50.3) and last (M = 50.7) evaluation, always remaining around the populational mean. It should be noted that this variable was measured using a task in which a maximum number of words that begin with the letter “p” must be named within one minute. As such, the activity is conditioned not only by the student’s knowledge of the phonology of the language, but also by the speed at which they can access the lexicon and name the words, in addition to the breadth and depth of the vocabulary that they possess. In measurements from the first evaluation, when the participants were at the beginning of their reading learning, they used shorter and more frequent words (such as pera [pear], papá [dad], etc.); however, during the second grade, they turned to a more complex vocabulary—to longer and less frequent words (such as panadero [baker], pintura [paint], pistacho [pistachio], etc.). Consequently, although the number of words did not greatly increase and scores from the first evaluation (M = 50.3) and the last (M = 50.71) are similar, one observes both a greater wealth of vocabulary and a potential difficulty in student performance relating to the time limit, since students take longer to produce the words and waste more seconds, even as they operate with a more complex lexicon [1,17].

With respect to the decoding variable, an improvement was found in the scores for both word reading and reading without meaning. This confirms the positive effect of an HLE (A) program on reading recognition, in accordance with the data found by Mora-Figueroa et al. (2016) and Niklas and Schneider (2014) [14,34]. In their respective studies, these researchers observed an improvement in reading decoding (identification of letters, and recognition of words and pseudowords) following the application of HLE (A) programs.

However, as in the case of phonological awareness (strongly related to reading recognition), there is a difference in the evolution of the two variables included for reading decoding. While the task used to measure word reading consisted of recognizing a list of words, that of reading without meaning involved participants reading a set of words and pseudowords that were inserted into a text. One may surmise that during the second year (7 to 8 years of age), when students were in the automation phase, they made more phonological errors, making greater use of the lexical route and relying on the narrative context. Thus, the improvement in the reading without meaning variable was greater during the first year and decreased during the second year. Ultimately, following the application of the HLE program (A), the scores (M = 56.94) were higher than those obtained before the intervention (M = 44.78).

It should also be noted that although this study does not present data on the performance of participants in reading comprehension, as it forms part of a larger investigation, we do know that, despite errors in reading accuracy, children were ultimately better able to understand. Between the ages of 7 and 8, as a result of the experience gained through the HLE (A) program, learning of the reading act was accelerated and students performed a less literal reading, as they were able to better reconstruct the general meaning of the text—as is characteristic of automated reading from the age of 8.

Part of this experience, in accordance with our hypothesis, was also reflected in a broader and richer lexicon. Thus, it is not surprising that improvements were found in the vocabulary variable subsequent to the application of the HLE (A) program, when comparing the scores obtained on the first and the last occasions of evaluation. In this regard, significant differences were observed (*p*-value < 0.05) both in semantic fluency, highly associated with the breadth of the lexicon, and in verbal comprehension, which is related to the depth of vocabulary. These results are consistent with those obtained in other studies wherein literacy skills and activities at home were found to correlate positively with vocabulary within the age range of 4 to 6 years [4,14].

Regarding the oral narrative comprehension variable, in line with our expectations, a significant change was detected (*p*-value < 0.05) following the application of the HLE (A) program. As such, it can be stated that the intervention had a positive effect on the former. The data obtained in this study following the initial 9 months of the HLE (A) program’s application are similar to those found by Mora-Figueroa et al. (2016) [34], who verified the efficacy of a family involvement program in improving oral comprehension in a group of children between 6 and 7 years old—the same age range as our sample subsequent to the first training period.

Based on the data obtained, it can thus be stated that the children improved their level of phonological awareness, reading decoding, vocabulary, and oral narrative comprehension as a result of the application of an HLE (A) program. The latter sought to promote a family literacy environment, through the involvement of parents in the program’s reading activities, allowing families to dedicate more time—on a more frequent basis—to having quality interactions with their children. Consequently, we hope that the intervention has had positive effects on children and parents’ affective relationships, although this remains under study, forming part of the research carried out.

Furthermore, given the increase in frequency and quality of interactions between parents and children, it can be assumed that children were increasingly aware of their family’s concern for their reading tasks and school learning, which can only have fostered their interest in reading and the act of reading. Because reading opens the doors to new information, an improvement and expansion of the reader’s knowledge—in addition to their motivation to read and learn—should be expected.

Further to this, it should be noted that all the variables measured in the study are considered skills that are fundamental to reading and that positively influence the reading ability of children, as indicated by the scientific literature on the subject [22,25,53,54]. Given the clear relationship between reading and writing, it can be expected that this would also apply to children’s writing performance.

In brief, it would be appropriate for this type of program to begin in the early childhood stage of education and continue into elementary education. This is because it not only helps students to strengthen their reading ability; it also accelerates their development and increases children’s interest in knowledge in general, has a positive impact on the linguistic and cognitive skills involved in the teaching-learning process, and benefits family and peer relationships. In fact, in the qualitative data observed during the course of the research, it was found that a topic of interest among the children at recess was reading (which books were being read, which were their favorites, etc.). In addition, the involvement and support from teachers within the program proved to be a source of motivation for parents and children, as they engaged in daily contact with families and promoted the application of the HLE (A) program.

### Limitations

In the course of the investigation, limitations were detected, such as the lack analysis on the type of family relationships and the absence of a control group, which could not be put in place due to the ethical reasons mentioned in the description of the sample.

With regard to future lines of research, it would be interesting to specifically analyze the quality and type of relationships established between parents and children. Although fidelity to treatment has been taken into account and compliance with the guidelines was monitored through the follow-up registers explained above, quantitative analyses that measure these variables could contribute to the enrichment of future studies. On the other hand, if they are to attend to the socio-economic and cultural variables related to the HLE [26,55]—such as the availability of resources or the type of literacy activities carried out at home—then participants from other educational centers may need to be included and a more heterogeneous and larger sample obtained. In this respect, it is to be noted that applying the HLE (A) program in a colegio concertado (charter school; state-funded but privately managed) with a middle- and upper middle-class demographic allowed us to control some variables (such as the parents’ low reading level, cultural differences, etc.) that, otherwise, would have required procedural modifications that are difficult to implement.

## 5. Conclusions

The present study suggests the importance of HLE (A) to achieving improvement in the psycholinguistic and cognitive skills fundamental to reading. Therefore, it may be assumed that the HLE (A) program will positively influence children’s reading performance and, probably, their writing performance and their learning at school. By attaining a greater command and knowledge of the elements that make up the language, a richer vocabulary, and a better understanding of oral narratives, not only will students more easily read and complete activities that include texts, statements, and written orders, they will also require less effort to understand teachers’ explanations and to express themselves verbally. To all these benefits may be added a strengthening of family relationships and an increased interest in the written code and, subsequently, the reading habit and school learning.

Although they were not specifically evaluated in this study, it is also expected that HLE (A) programs improve affective relationships, by increasing the quantity and quality of interactions, establishing a greater number of moments of enjoyment within the family environment, and giving rise to the creation and/or knowledge of ideas, thoughts, and common interests, etc. All of these represent factors that could have an impact on a child’s motivation for tasks related to the written code.

The practical implications of this study are of great importance, as they give a clear indication that the improvement of these variables depends not only on the educational methodology applied in school, but also on the context and environment in which a child develops, especially during the first years of life and the initial years of formal schooling. In this respect, the promotion of HLE (A) intervention programs will benefit children’s learning—specifically, the acquisition and development of skills related to reading.

In summary, the present study has shown that the HLE (A) program, focused on assisting families in carrying out reading tasks with their children, helps to improve psycholinguistic and cognitive variables. Moreover, it is not only useful in supporting family relationships and in accelerating reading learning, but, in view of the requirements of the reading process, the HLE (A) program constitutes a means to improve the cognitive development of children.

## Figures and Tables

**Table 1:** Overview of the instruments used in the study.

Variable	Instrument	Task
Phonological awareness	DST-J	Phonemic segmentation, rhymes, and verbal fluency
Reading decoding	DST-J	Reading and reading without meaning
Vocabulary	DST-J	Semantic fluency and vocabulary
	WISC-V	Verbal Comprehension Index: Similarities and Vocabulary Subtests
Oral narrative comprehension	PROLEC-R	Oral comprehension

**Table 2:** Summary of *p*-values for the Type III sum of squares.

Variables		Group Factor *p*-Value	Experimental Factor *p*-Value	Interaction between Factors *p*-Value
**Phonological awareness**	**Verbal fluency**	0.2054	0.0172	0.6292
	**Rhymes**	0.4831	0.0000	0.6436
	**Phonetic segmentation**	0.8652	0.0000	0.5783
**Reading decoding**	**Word reading**	0.8144	0.0000	0.1635
	**Reading without meaning**	0.7403	0.0000	0.2145
**Vocabulary**	**Semantic fluency**	0.5367	0.0101	0.5861
	**Verbal comprehension**	0.3717	0.0012	0.3261
**Oral narrative comprehension**	**Oral narrative comprehension**	0.8071	0.0000	0.0228

Note: After confirming through the analysis of variance that the model was significant in all cases, we studied and checked why this was, by means of type III sum of squares. If *p*-value < 0.05, there are significant differences between the mean responses in the levels of the effect indicated at the 5% level of significance. Such as could be observed, there was only a slight interaction between the groups and the experimental factor in the variable oral narrative comprehension. This means that the improvement of one group is slightly greater than that of the other, but there is no significant difference. Therefore, the evolution of both groups continues to be considered similar, being equally affected by the intervention.

**Table 3:** Means of minimum squares for each variable (95% confidence intervals).

**Phonological awareness**	**Rhymes percentile scores**	**Evaluation**	**Mean**	**Standard Error**	**95% Confidence Interval**	
		1	30.0577	2.65973	24.8023	35.3131
		2	45.5192	2.65973	40.2639	50.7746
		3	39.0769	2.65973	33.8215	44.3323
		4	53.0	2.65973	47.7446	58.2554
	**Verbal fluency percentile scores**	**Evaluation**	**Mean**	**Standard error**	**95% Confidence interval**	
		1	50.3077	3.17176	44.0406	56.5748
		2	63.0769	3.17176	56.8098	69.344
		3	55.0962	3.17176	48.829	61.3633
		4	50.7115	3.17176	44.4444	56.9787
	**Phonemic segmentation percentile scores**	**Evaluation**	**Mean**	**Standard error**	**95% Confidence interval**	
		1	55.0769	2.52057	50.0965	60.0573
		2	75.4808	2.52057	70.5004	80.4612
		3	69.9808	2.52057	65.0004	74.9612
		4	61.3462	2.52057	56.3657	66.3266
**Reading decoding**	**Word reading percentile scores**	**Evaluation**	**Mean**	**Standard error**	**95% Confidence interval**	
		1	35.9074	2.13549	31.6892	40.1256
		2	54.2963	2.13549	50.0781	58.5145
		3	51.963	2.13549	47.7448	56.1812
		4	60.2778	2.13549	56.0596	64.496
	**Reading without meaning percentile scores**	**Evaluation**	**Mean**	**Standard error**	**95% Confidence interval**	
		1	44.7778	2.38512	40.0665	49.4891
		2	65.8148	2.38512	61.1035	70.5261
		3	69.963	2.38512	65.2516	74.6743
		4	56.9444	2.38512	52.2331	61.6558
**Vocabulary**	**Semantic fluency percentile scores**	**Evaluation**	**Mean**	**Standard error**	**95% Confidence interval**	
		1	45.4038	3.34417	38.7961	52.0116
		2	56.8077	3.34417	50.1999	63.4155
		3	46.5769	3.34417	39.9691	53.1847
		4	57.9615	3.34417	51.3538	64.5693
	**Verbal comprehension fluency percentile scores**	**Evaluation**	**Mean**	**Standard error**	**95% Confidence interval**	
		1	59.3077	2.56274	54.2439	64.3714
		2	72.3269	2.56274	67.2632	77.3907
		3	65.9423	2.56274	60.8786	71.0061
		4	71.6538	2.56274	66.5901	76.7176
**Oral narrative comprehension**	**Oral narrative comprehension direct scores**	**Evaluation**	**Mean**	**Standard error**	**95% Confidence interval**	
		1	2.74	0.182726	2.37883	3.10117
		2	4.08	0.182726	3.71883	4.44117
		3	3.74	0.182726	3.37883	4.10117
		4	4.76	0.182726	4.39883	5.12117

## Data Availability

The data of this study are not public, in order to respect the confidentiality of the participants and their legal guardians, since the sample is composed of minors.
